# Parotid small cell carcinoma presenting with long-term survival after surgery alone: a case report

**DOI:** 10.1186/1752-1947-6-431

**Published:** 2012-12-28

**Authors:** Takeharu Kanazawa, Noriyoshi Fukushima, Hidetaka Tanaka, Juntaro Shiba, Hiroshi Nishino, Hiroyuki Mineta, Keiichi Ichimura

**Affiliations:** 1 Department of Otolaryngology/Head and Neck Surgery, Jichi Medical University School of Medicine, 3311–1, Shimotsuke, 329-0498, Japan; 2 Department of Pathology, Jichi Medical University School of Medicine, Shimotsuke, 329-0498, Japan; 3 Department of Otolaryngology/Head and Neck Surgery, Hamamatsu University School of Medicine, Hamamatsu, 431-3192, Japan

## Abstract

**Introduction:**

Primary involvement of the salivary glands in small cell carcinoma is rare, and has one of the worst prognoses of salivary gland neoplasms. However, it has been reported that some cases have a favorable outcome, although the prognostic factors are still under consideration. Multidisciplinary therapy was usually required to achieve long-term survival. Recently, a resemblance of some small cell carcinomas of the salivary gland to cutaneous Merkel cell carcinoma was suggested; the latter have the potential for spontaneous regression, which is related to a favorable clinical outcome.

**Case presentation:**

We present a locoregional advanced parotid small cell carcinoma with multiple lymph node metastases in an 87-year-old Asian woman. The tumor was controlled by surgery alone, and nine-year disease-free survival was achieved without any adjunctive therapy. To the best of our knowledge, this is the longest reported follow-up of head and neck small cell carcinoma.

**Conclusion:**

We believe this to be the first case of small cell carcinoma with involvement of the salivary glands reported in the literature with a good outcome after surgery alone without any adjunctive therapy.

## Introduction

Primary involvement of the salivary glands in small cell carcinoma (SmCC) is rare, and tumors in the salivary glands account for less than 1% of all carcinomas of the parotid gland and 3.5% of all malignant tumors of minor salivary glands. This tumor has one of the worst prognoses of salivary gland neoplasms. The prognosis for patients with SmCC of the salivary glands has been reported to be more favorable than for those with SmCC of other sites [1-4]. However, there is no doubt that parotid SmCC is a high-grade malignancy that should be treated aggressively. Surgery, adjunctive radiation therapy and/or chemotherapy have been performed in most cases [1].

SmCCs of the salivary gland are classified into neuroendocrine types and ductal carcinomas. The neuroendocrine type can be classified further into Merkel-cell-like SmCC and pulmonary variants, based on cytokeratin 20 immunoreactivity with a dot-like staining pattern [2]. Recently, the resemblance of Merkel-cell-like SmCCs of salivary gland to cutaneous Merkel cell carcinoma has been suggested, and some of them have spontaneous regression potential related to a favorable clinical outcome [1,2]. In this article, we report a locoregional advanced parotid SmCC that had an unusual clinical course. The tumor was controlled by surgery alone, and nine-year disease-free survival was achieved without any adjunctive therapy. We also studied the expression of an oncogene or tumor suppressor gene, and demonstrated the expression of *mammary serine protease inhibitor* (*Maspin*), which is an important tumor suppressor gene of salivary gland carcinomas.

## Case presentation

An 87-year-old Asian woman in good general health presented with a progressively enlarging mass located in her left preauricular region. Fine needle aspiration (FNA) cytology analysis performed in the clinic suggested malignant cells of uncertain origin, but the tumors showed a rapid and consistent regression after FNA. During six months of observation, the tumor regrew and the patient was referred to our hospital.

**Figure 1:**
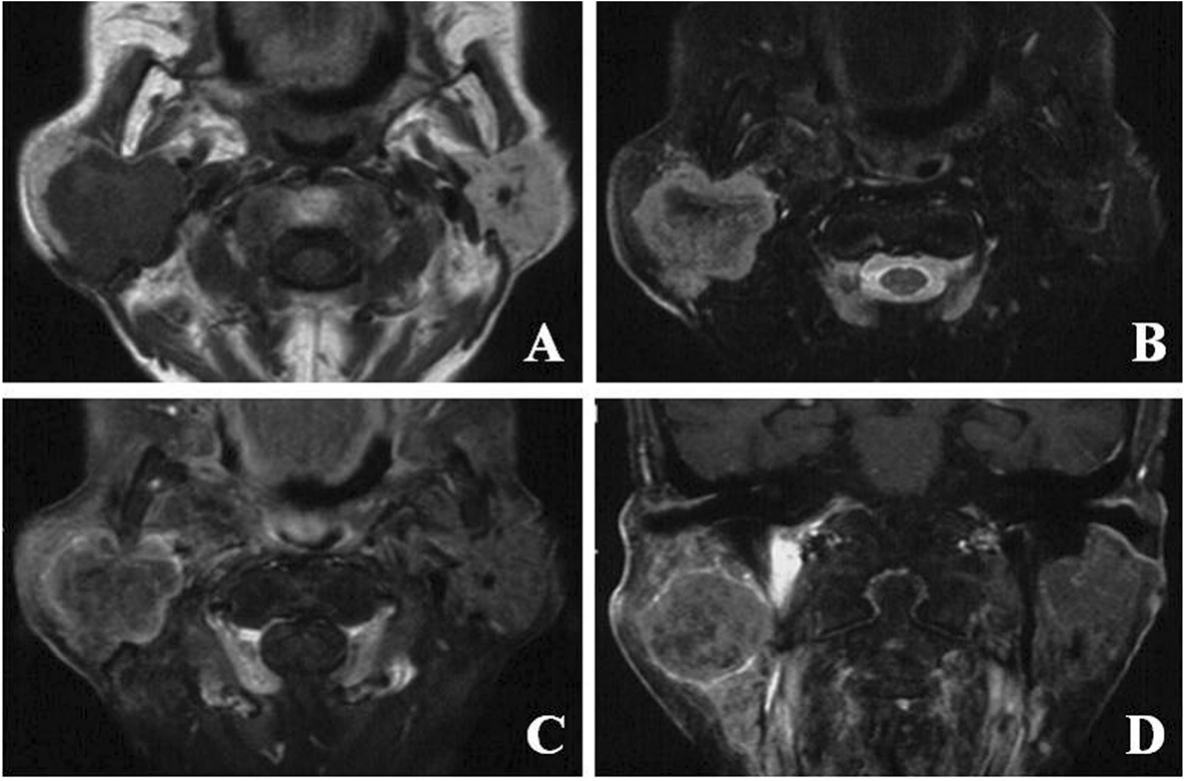
**Magnetic resonance imaging scans.** Magnetic resonance imaging shows this lesion to be clearly circumscribed, with **(A)** homogeneous hypo-iso signal intensity on T1-weighted images and **(B)** heterogeneous high signal intensity on T2-weighted images. **(C)** Axial and **(D)** coronal gadolinium-enhanced T1-weighted images show the presence of a circumscribed and heterogeneously enhanced tumor.

A physical examination revealed a 55-mm tumor located in the parotid with extension to the retroparotid area, and swelling of her left laterocervical lymph node. A computed tomography scan showed a peripheral enhanced mass in her right parotid gland. Magnetic resonance imaging revealed low signal intensity on T1-weighted images and iso-high signal intensity on T2-weighted images. The images showed the lesion to be clearly circumscribed, with homogeneous hypo-iso signal intensity on T1-weighted images and heterogeneous high signal intensity on T2-weighted images. The tumor demonstrated circumscribed and heterogeneous enhancement on gadolinium-enhanced T1-weighted images (Figure 1). Computed tomography scans of her thorax and abdomen did not reveal involvement of other sites.

**Figure 2:**
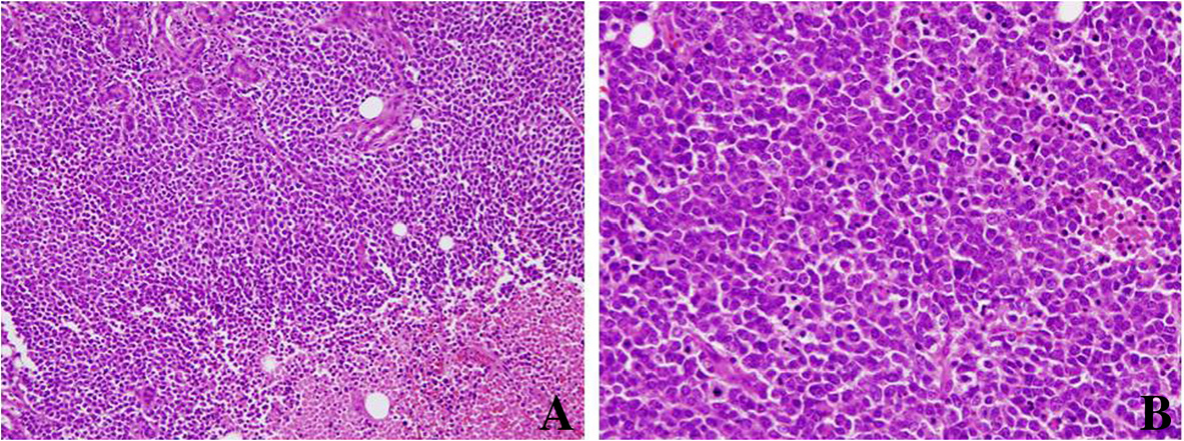
**Histological features. (A)** Diffuse growth with necrosis (lower right) of tumor cells is seen. Several residual ducts and glands are observed in the tumor. **(B)** The tumor shares the features of small cell carcinomas seen in other organs. It is composed of small- to medium-sized epithelioid cells with hyperchromatic, finely granular nuclei and scant cytoplasm. Mitotic figures are frequently seen.

**Figure 3:**
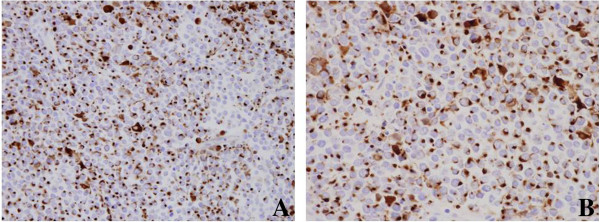
**Immunohistochemistry for cytokeratin 20.** Most tumor cells express cytokeratin 20 with a characteristic dot-like pattern. **(A)** Original magnification ×400; **(B)** original magnification ×600.

FNA was performed again and a cytological examination revealed a Papanicolaou classification of Class V, suggesting SmCC. A total parotidectomy and modified neck dissection were performed. On histopathology, the tumor showed diffuse growth with confluent necrosis in the salivary gland. The tumor cells had scant cytoplasm and hyperchromatic nuclei without prominent nucleoli. Mitotic figures were frequently observed (Figure 2). Neoplasmic elements reveal positivity for cluster of differentiation 56, neuron specific enolase, synaptophysin and a dotted staining pattern with cytokeratin 20 (Figure 3). Based on our histopathological findings, a diagnosis of a Merkel-cell-like SmCC of the parotid gland was made. Additional studies were positive for *Maspin*.

Our patient’s postoperative course was uneventful, and no postoperative radiotherapy was administered. The nine-year clinical follow-up, which is the longest follow-up of head and neck SmCC in our knowledge, revealed no locoregional recurrence or distant metastasis.

## Discussion

The parotid gland tumor in our case appeared on histology to be a SmCC. No tumor formation was recognized in other locations; therefore, this was an extremely rare primary SmCC of the parotid gland. This case also has the longest follow-up to the best of our knowledge. SmCC can occur in any organ, although the vast majority occur in the lung. The prognosis for patients with SmCC of the salivary glands has been reported to be more favorable than for those with SmCC of the lung or larynx [1,2,4]. However, there is no doubt that parotid SmCC is a high-grade malignancy that should be treated aggressively. The main treatment for parotid SmCC is a surgical approach with partial or total parotidectomy. The association of radiotherapy with surgery has shown a decrease in relapses and an increase in survival. Seventy-five percent of local relapses occurred in cases where surgery had been the only treatment, whereas, when associated with radiotherapy, the rate of local relapse was 20% [1,5]. Our case was controlled by surgery alone, and nine-year disease-free survival was achieved without any adjunctive therapy despite the advanced nature of the disease.

Recently, two parotid SmCCs with unusual clinical courses were reported. Mulder *et al*. presented a primary SmCC of the parotid with massive local recurrence that regressed spontaneously [6]. Jorcano *et al*. also presented an advanced case where a complete response and long-term survival was achieved by radiotherapy alone [5]. Both cases involved Merkel-cell-like SmCC. The neuroendocrine type of SmCC can be subdivided into Merkel-cell-like and pulmonary varieties on the basis of cytokeratin 20 immunoreactivity with a dot-like staining pattern [7]. It has been speculated that some salivary Merkel-cell-like SmCC are closely related biologically to cutaneous Merkel cell carcinoma, which is known to be less aggressive than extrasalivary SmCC [8]. One possibility for a favorable clinical outcome after initial treatment is the potential for spontaneous regression. We could find one only case of spontaneous regression of a salivary Merkel-cell-like SmCC [6], but spontaneous regression of cutaneous Merkel cell carcinomas has been described in 20 cases [9,10]. Furthermore, 10% to 20% of all cases of metastasized cutaneous Merkel cell carcinomas present no obvious primary tumor [10]. It is not known if the primary lesion had regressed in these cases. The reasons for spontaneous regression remain unclear. Mulder *et al*. explained the mechanism of spontaneous regression as follows: “apoptotic events seem to play an important part, and a local T-cell mediated immune response triggered by surgical trauma might also be involved”. The hypothesis that spontaneous regression occurs after surgery might help understand this unusual clinical course. The fact that spontaneous regression was observed after initial FNA also supports this hypothesis in our case.

To understand the mechanism of spontaneous regression further, we studied the expressions of various oncogenes or tumor suppressor genes, and demonstrated the expression of *Maspin*. *Maspin* belongs to the serine protease inhibitor family and may be associated with a favorable prognosis in common salivary gland carcinomas such as adenoid cystic carcinoma, mucoepidermoid carcinoma and carcinoma ex pleomorphic adenoma [11]. Although *Maspin* expression in salivary SmCC might be important information, the relationship between *Maspin* and spontaneous regression is under consideration.

## Conclusion

The mechanism of spontaneous regression in salivary SmCC is not fully understood, but our experience with this case leads us to suggest that some cases have a favorable clinical outcome that is related to the potential for spontaneous regression.

### Consent

Written informed consent was obtained from the patient’s guardian for publication of this case report. A copy of the written consent is available for review by the Editor-in-Chief of this journal.

## Competing interest

The authors declare that they have no competing interests.

## Authors’ contributions

TK presented the case history, researched the topic and helped draft the manuscript. NF provided pathologic diagnosis. JS reviewed the literature and drafted the manuscript. HT and HN performed the surgery. HM and KI were the supervising consultants who obtained consent for publication and edited the manuscript. All authors read and approved the final manuscript.
